# An Aggravated Trajectory of Depression and Anxiety Co-morbid with Hepatitis C: A Within-groups Study of 61 Australian Outpatients

**DOI:** 10.2174/1745017901511010174

**Published:** 2015-11-13

**Authors:** Benjamin J.R. Stewart, Deborah Turnbull, Antonina A. Mikocka-Walus, Hugh Harley, Jane M. Andrews

**Affiliations:** 1 School of Psychology, University of Adelaide, Australia;; 2 Department of Health Sciences, University of York, United Kingdom;; 3 Department of Gastroenterology and Hepatology, Royal Adelaide Hospital, Australia;; 4 Discipline of Medicine, University of Adelaide, Australia

**Keywords:** Anxiety, depression, hepatitis C, prognosis, trajectory.

## Abstract

*Background*: This study aimed to explore the course of depression and anxiety in chronic hepatitis C patients. *Methods*: Data were combined from two studies: (1) Hospital Anxiety and Depression Scale (HADS) scores in 395 consecutive Australian outpatients from 2006 to 2010 formed the baseline measurement; and (2) Depression Anxiety Stress Scales (DASS) scores in a survey of a sub-sample of these patients in 2011 formed the follow-up measurement. After converting DASS to HADS scores, changes in symptom scores and rates of case-ness (≥8), and predictors of follow-up symptoms were assessed. *Results*:  Follow-up data were available for 61 patients (70.5% male) whose age ranged from 24.5 to 74.6 years (M=45.6). The time to follow-up ranged from 20.7 to 61.9 months (M=43.8). Baseline rates of depression (32.8%) and anxiety (44.3%) increased to 62.3% and 67.2%, respectively. These findings were confirmed, independent of the conversion, by comparing baseline HADS and follow-up DASS scores with British community norms. Baseline anxiety and younger age predicted depression, while baseline anxiety, high school non-completion, and single relationship status predicted anxiety. *Conclusion*: ** **This study demonstrated a worsening trajectory of depression and anxiety. Further controlled and prospective research in a larger sample is required to confirm these findings.

## INTRODUCTION

Psychiatric co-morbidity is prevalent in chronic hepatitis C [CHC; 1] and results in diminished quality of life [2], increased fatigue [3, 4] and pain [5], and impaired anti-viral treatment outcomes [6]. It appears that CHC itself may be particularly associated with poorer mental health outcomes, with research showing that the rate of major depression was higher in CHC patients compared with controls or chronic hepatitis B patients [7]. Psychosocial stressors are a contributing factor to this morbidity, and include the adverse effect of diagnosis, anti-viral treatment, stigma, and fears regarding disease progression or viral transmission [8]. Research has demonstrated poorer quality of life in people aware of their CHC infection compared with those unaware [9, 10]. Thus, the ability of patients to adjust to, and cope with, psychosocial stressors accompanying and following the diagnosis of CHC may be critical in determining the longitudinal course of psychiatric disorders. However, little is known about the course of psychiatric co-morbidity in this cohort. This study aimed to assess the course of depression and anxiety symptoms in a cohort of South Australian CHC outpatients of the Royal Adelaide Hospital (RAH) liver clinic.

## MATERIALS AND METHODOLOGY

### Design and Participants

This within-subjects study combined and compared data collected on a sub-set of CHC outpatients from two previous studies. In the first [11], Hospital Anxiety and Depression Scale [HADS; 12] scores, collected as part of standard clinical care at appointments at the RAH liver clinic from 2006, were analysed to explore the prevalence and predictors of depression and anxiety in 395 consecutive CHC outpatients from 2006-2010. In the second [13], CHC outpatients from this clinic completed a postal survey in late 2011 and early 2012 exploring psychological treatment acceptability, including a measurement with the Depression Anxiety Stress Scales [14]. Data available for participants of both studies (n=61) were collated to assess the level of depression and anxiety at the two points of assessment.

### Procedure

A recent study facilitated a method of converting scores between the HADS and DASS. Covic and colleagues [15] measured depression and anxiety in British and Australian Rheumatoid Arthritis (RA) patients using the HADS and the DASS. Through use of Rasch Analysis, they were able to calibrate the two scales by mapping scores on to a common underlying metric of psychopathology. The present study applied this metric to convert DASS scores at follow-up in 2011 to HADS scores. A cut-off score ≥ 8 on the HADS was used to determine depression or anxiety, in accordance with recommendations [16, 17]. The ethics committees of the RAH and University of Adelaide provided approval for the two studies comprising it the data for this paper. This research was conducted in accordance with the Declaration of Helsinki.

### Analysis

Differences between HADS scores at baseline (T1) and converted HADS scores at follow-up (T2) were compared using repeated samples t-tests. Rates of case-ness at T1 and T2 were compared using McNemar’s test. Levels of depression and anxiety were compared against British norms for T1 HADS scores [18] and T2 DASS scores [19], and the discrepancy between effect sizes analysed, in order to provide an assessment of change independent of DASS conversion. Univariate associations with T2 HADS scores were conducted using Pearson’s correlation for continuous predictors and independent samples t-tests for categorical predictors. Multivariate associations were tested using linear regression models with hierarchical entry of predictors with a univariate association of p<0.05, with baseline depression and anxiety scores entered at Step 1, and all other predictors at Step 2.

## RESULTS

Socio-demographic and medical data is presented in Table **1**. Of the 61 participants for whom T1 and T2 data was available, 43 (70.5%) were male. Their age ranged from 24.49 to 74.61 years (M=45.61, SD=10.08) and the time between T1 and T2 assessments ranged from 20.64 to 61.92 months (M=43.80, SD=12.24).

As shown in Table **2**, depression and anxiety rates increased by T2. The odds of developing new depressive case-ness by T2 was 10 times higher than the odds of T1 cases going into remission from depression (p<0.001, 95% CI:2.34-42.78). Similarly, the odds of developing anxiety case-ness by T2 was 4.5 times higher than the odds of remission from anxiety by T2 (p=0.004, 95% CI:1.52-13.30). Finally, the odds of developing co-morbidity by T2 was 4 times higher than the odds of remission from co-morbidity by T2 (p=0.004, 95% CI:1.50-10.66).

As shown in Fig. (**1**), the sample as a whole experienced a significant increase in both depression (*t*(60)=6.41, *p*<0.001, *d*=0.82) and anxiety (*t*(60)=4.08, *p*<0.001, *d*=0.52) from T1-T2. When analysed based on case-ness at T1, depression scores increased significantly in patients without baseline case-ness (*t*(31)=6.28, *p*<0.001,* d*=1.15) and with one baseline disorder (*t*(10)=2.87, *p*=0.017, *d*=0.88) but not in those with T1 co-morbidity (*t*(17)=1.64, *p*=0.120, *d*=0.39). Anxiety scores increased significantly in those without baseline case-ness (*t*(31)=5.24, *p*<0.001, *d*=1.01) and remained stable in those with one baseline disorder (*t*(10)=0.75, *p*=0.473, *d*=0.23) or T1 co-morbidity (*t*(17)=0.53, *p*=0.605, *d*=0.13).

As shown in Fig. (**2**), at T1 the present sample was significantly disadvantaged and was compared to British community HADS norms with respect to both depression (*t*(1851)=4.03, *p*<0.001, Cohen’s *d*=0.46) and anxiety (*t*(1851)=2.43, *p*=0.015, Cohen’s *d*=0.29). When DASS scores were compared to British norms at T2, this discrepancy had widened markedly for both depression (*t*(1853)=11.83, *p*<0.001, Cohen’s *d*=1.15) and anxiety (*t*(1853)=12.44, *p*<0.001, Cohen’s *d*=1.16).

Univariate predictors of depression and anxiety at T2 were then assessed, including age at T1, gender, nationality, education, the Socio-Economic Index For Areas (SEIFA) relative socio-economic advantage and disadvantage index based on post-code areas at T1 [20], relationship status at T1, previous injecting drug use (IDU), years since diagnosis at T2, the time between T1 and T2 assessments, anti-viral treatment between T1 and T2, achieving an SVR between T1 and T2, and T1 anxiety and depression. Depression and anxiety at T1 were significantly and positively correlated with both depression and anxiety scores at T2, while age at T1 was negatively correlated with depression scores at T2. Those who had completed high school and those who were in a relationship at T1 had significantly lower depression and anxiety scores at T2.

Significant univariate predictors were then entered into the multivariate analysis, as shown in Table **3**. On step 1, T1 depression and anxiety scores were entered. Only anxiety was at T1 independently predicted both depression and anxiety scores at T2. At step 2, education and relationship status were entered into both models and age was added to the depression model. Age remained significant and explained an additional 11% of the variance in depression scores at T2, while education and relationship status remained significant and explained an additional 17% of the variance in anxiety at T2.

## DISCUSSION

This study demonstrated a poor trajectory of depression and anxiety in which four groups could be identified: (1) those who were non-cases at both baseline and follow-up (23.0%); (2) those who were non-cases at baseline, but whose symptoms increased to case-ness thresholds by follow-up (29.5%); (3) those who were cases at baseline and follow-up (45.9%); and (4) the sole individual who was a case at baseline and recovered by follow-up (1.6%). Two studies have also reported worsening in symptoms over time in Crohn’s disease [21] and cardiovascular patients [22]. However, these findings contradict the stability reported over one year in two smaller studies with CHC [23, 24]. Moreover, research in other populations have reported stability over time, including in the general population [25] and those with HIV [26, 27], irritable bowel syndrome and inflammatory bowel disease [24, 28], cardiovascular disease [29, 30], and RA [31, 32].

It is possible that the aggravated course observed here can be explained by the differential nature of CHC and the psychosocial stressors it poses and/or the nature of the populations that typically acquires CHC – comprising mostly current or former IDUs (67% in this cohort). Also, previous research has reported a worse prognosis for those with co-morbid depression and anxiety [33, 34], which is common in CHC patients [1] and, particularly, in the cohort from which this sample was drawn [11].

However, this does not account for the aggravation in those without morbidity at baseline - the group in which the main symptom increase occurred. The correlation between depression and anxiety scores at baseline provides insight into the general level of co-morbid symptomatology in the sample regardless of case-ness. In the British community, the correlation between HADS anxiety and depression scores is moderate (*r*=0.53, *p*<0.001). In the cohort from which the present sample was drawn [11], the correlation is significantly higher (*r*=0.66, Z=3.63, *p*<0.001), indicating a higher degree of co-morbid symptoms in this patient group.

Interestingly, time since diagnosis was not related to depression or anxiety at follow-up. There was also no association between the change in psychopathology and the variable time difference between the baseline and follow-up assessments across patients. This suggests that the aggravation in symptoms being observed may not be stemming from a failure and to adjust with the diagnosis of CHC per se or from the mere passage of time. It is possible that the progression observed reflects a difficulty in adjusting to new psychosocial stressors, such as disease progression and treatment considerations. These stressors do not occur at predictable time points following diagnosis, as CHC can progress quite slowly and patients may present for specialist treatment at different times following diagnosis.

In the multivariate analysis, baseline anxiety, but not depression, remained an independent predictor of both increased depression and anxiety at follow-up. This is supported by longitudinal community-based studies which have found that anxiety leads to depression more often than the reverse [33, 35]. After accounting for baseline depression and anxiety, age remained a significant predictor of decreased depression at follow-up, while education and relationship status remained independent predictors of decreased anxiety - consistent with other research [25, 36]. 

## LIMITATIONS

This study has a number of limitations. There were no control comparison subjects and the length of follow-up varied, due to this studies post-hoc use of routinely collected clinical data as the baseline measurement. While the present cohort was compared with normative data.The sample size was too small to match the age and gender which can influence the expression of mood and anxiety symptoms. Data was not available for whether patients were diagnosed with specific disorders. Symptoms of depression and anxiety can be present across varying mood and anxiety disorders as well as in other psychiatric illnesses including personality and psychotic disorders. It was also not possible to analyse previous or current psychiatric treatment. The sample was small and the participants of the second study who provided follow-up data were self-selected [13], introducing the possibility of sample biases. However, excluding a slightly lower response rate in previous recipients of anti-viral treatment (16% vs. 25%), there were no differences between survey responders and non-responders [13]. Critically, there were no differences in HADS scores. Thus, both non-responders and responders to the survey experienced comparable mental health at baseline. However, it is possible those who experienced a worse course of depression and anxiety after that baseline measurement were more inclined to respond at follow-up because the issue was more personally salient. The multivariate analysis should especially be regarded with caution due to the small sample size and relatively large number of predictor variables are used.

 The procedure of converting DASS scores at follow-up with HADS scores to compare symptoms rely on the assumption that the calibration of DASS and HADS scores by Covic and colleagues [15] is robust and equivalent between RA and CHC patients. To verify the findings independent of this conversion and its assumptions, baseline HADS and follow-up DASS scores were compared separately to British norms for the HADS [18] and DASS [19], respectively. At baseline, the present sample had significantly worse HADS scores than British norms. However, by follow-up this disadvantage, in comparison to British norms for the DASS, had inflated by a factor of 2.5 for depression and 4 for anxiety. 

Finally, subjects who achieved a SVR between baseline and follow-up assessments were included, as many receive ongoing care to assess for viral relapse and manage existing liver damage. If the SVR rates of the sample that provided follow-up data were lower than normal, this could explain their poorer mental health. However, of the 31 patients who received anti-viral treatment, 58% achieved a SVR, consistent with rates in those treated with interferon and ribavirin [37]. Moreover, SVR was not associated with follow-up depression or anxiety in this study. 

## CONCLUSION

This study found a high rate of co-morbid depression and anxiety which increased markedly over a period of up to five years in a small sample of Australian CHC outpatients. Future research would benefit from a controlled, prospective analysis in a larger sample, involving multiple assessments of symptoms and a focus on potential intervening variables such as psychiatric treatment, social support, and changes in CHC to related psychosocial stressors.

## Figures and Tables

**Fig. (1):**
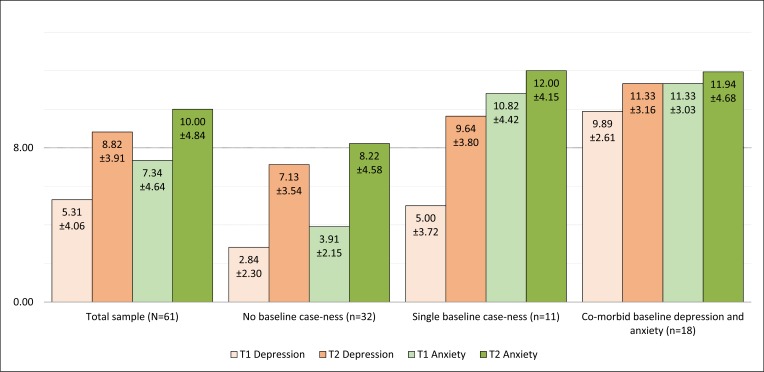


**Fig. (2):**
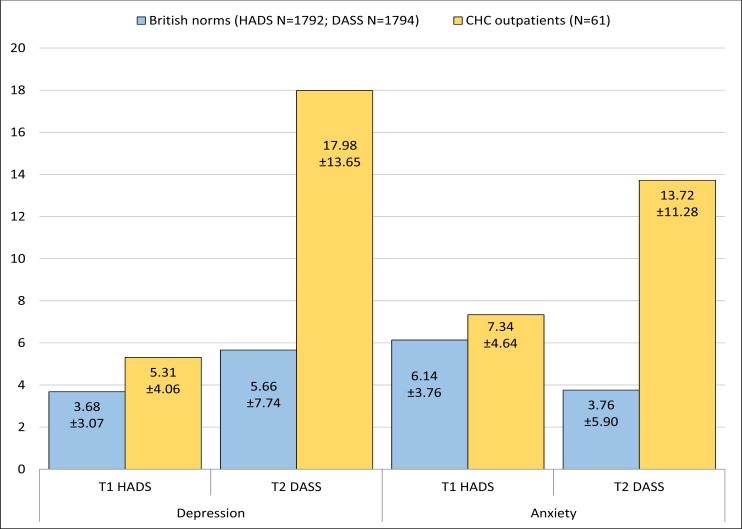


**Table 1.** Socio-demographic and medical characteristics of patients.

Categorical variables		n	%
Gender	Male	43	70.5
	Female	18	29.5
Education	Non-high school completer	28	45.9
	High school completer	33	54.1
Relationship status	Not partnered	42	73.7
	Partnered	19	26.3
Nationality	Born in Australia	44	72.1
	Born overseas	17	27.9
Previous IDU	No	20	32.8
	Yes	41	67.2
Anti-viral treatment from T1 to T2	No	30	49.2
	Yes	31	50.8
SVR from T1 to T2	No	43	70.5
	Yes	18	29.5
Continuous variables	Range	M	SD
Age (years)	24.49 – 74.61	45.61	10.08
SEIFA	807 – 1098	952.48	77.52
Time since diagnosis (years)	1 – 40	12.51	8.07
T1 to T2 interval (months)	20.70 – 61.93	43.78	12.21

IDU=Injecting drug use, SEIFA=Socio-Economic Index For Areas Index of Advantage/Disadvantage, based on post-code areas.

**Table 2.** Depression and anxiety case-ness rates at T1 and T2.

Case-ness type	Number of cases † (%)	
	Baseline	Follow-up
Depression‡	20 (32.8)	38 (62.3)
Depression alone	2 (3.3)	5 (8.2)
Anxiety‡	27 (44.3)	41 (67.2)
Anxiety alone	9 (14.8)	8 (13.1)
Any disorder	29 (47.5)	46 (75.4)
One disorder	11 (18.0)	13 (21.3)
Co-morbid disorders	18 (29.5)	33 (54.1)

† Cases are based on HADS subscale scores ≥ 8 ‡ Cases with ‘Depression’ or ‘Anxiety’ may also exhibit case-ness of the other disorder type, as compared with ‘Depression alone’ or ‘Anxiety alone’ wherein these cases only exhibit case-ness of that disorder type.

**Table 3.** Multivariate analyses of T2 depression and anxiety.

Variable		T2 Depression			T2 Anxiety		
		*b*	*SE b*	*β*	*b*	*SE b*	*β*
Step 1	*Constant*	*5.64*	*0.87*	* *	*6.61*	*1.10*	
	T1 Depression	0.15	0.15	.16	0.03	0.19	.03
	T1 Anxiety	0.32	0.13	.38*	0.41	0.16	.40*
Step 2	*Constant*	*10.10*	*3.42*	* *	*6.52*	*3.07*	
	T1 Depression	0.11	0.14	.11	-0.10	0.17	-.08
	T1 Anxiety	0.29	0.12	.35*	0.42	0.15	.42**
	Age	-0.09	0.04	-.23*	-	-	-
	High school completion	-1.59	0.90	-.20	-2.90	1.10	-.30*
	Relationship status	1.56	1.01	.18	3.03	1.23	.28*

## References

[1] el-Serag H.B., Kunik M., Richardson P., Rabeneck L. (2002). Psychiatric disorders among veterans with hepatitis C infection.. Gastroenterology.

[2] Gutteling J.J., Duivenvoorden H.J., Busschbach J.J., de Man R.A., Darlington A.S. (2010). Psychological determinants of health-related quality of life in patients with chronic liver disease.. Psychosomatics.

[3] Dwight M.M., Kowdley K.V., Russo J.E., Ciechanowski P.S., Larson A.M., Katon W.J. (2000). Depression, fatigue, and functional disability in patients with chronic hepatitis C.. J. Psychosom. Res..

[4] McDonald J., Jayasuriya J., Bindley P., Gonsalvez C., Gluseska S. (2002). Fatigue and psychological disorders in chronic hepatitis C.. J. Gastroenterol. Hepatol..

[5] Morasco B.J., Huckans M., Loftis J.M., Woodhouse J., Seelye A., Turk D.C., Hauser P. (2010). Predictors of pain intensity and pain functioning in patients with the hepatitis C virus.. Gen. Hosp. Psychiatry.

[6] Leutscher P.D., Lagging M., Buhl M.R., Pedersen C., Norkrans G., Langeland N., Mørch K., Färkkilä M., Hjerrild S., Hellstrand K., Bech P., NORDynamIC Group (2010). Evaluation of depression as a risk factor for treatment failure in chronic hepatitis C.. Hepatology.

[7] Carta M.G., Hardoy M.C., Garofalo A., Pisano E., Nonnoi V., Intilla G., Serra G., Balestrieri C., Chessa L., Cauli C., Lai M.E., Farci P. (2007). Association of chronic hepatitis C with major depressive disorders: irrespective of interferon-alpha therapy.. Clin. Pract. Epidemol Ment. Health.

[8] Stewart B.J., Mikocka-Walus A.A., Harley H., Andrews J.M. (2012). Help-seeking and coping with the psychosocial burden of chronic hepatitis C: a qualitative study of patient, hepatologist, and counsellor perspectives.. Int. J. Nurs. Stud..

[9] Dalgard O., Egeland A., Skaug K., Vilimas K., Steen T. (2004). Health-related quality of life in active injecting drug users with and without chronic hepatitis C virus infection.. Hepatology.

[10] Rodger A.J., Jolley D., Thompson S.C., Lanigan A., Crofts N. (1999). The impact of diagnosis of hepatitis C virus on quality of life.. Hepatology.

[11] Stewart B., Mikocka-Walus A., Morgan J., Colman A., Phelps M., Harley H., Andrews J. (2012). Anxiety and depression in Australian chronic hepatitis C outpatients: prevalence and predictors.. Australas. Psychiatry.

[12] Zigmond A.S., Snaith R.P. (1983). The hospital anxiety and depression scale.. Acta Psychiatr. Scand..

[13] Stewart B.J., Turnbull D., Mikocka-Walus A.A., Harley H.A., Andrews J.M. (2013). Acceptability of psychotherapy, pharmacotherapy, and self-directed therapies in Australians living with chronic hepatitis C.. J. Clin. Psychol. Med. Settings.

[14] Lovibond S.H., Lovibond P.F. (1995). Manual for the depression anxiety stress scales..

[15] Covic T., Cumming S.R., Pallant J.F., Manolios N., Emery P., Conaghan P.G., Tennant A. (2012). Depression and anxiety in patients with rheumatoid arthritis: prevalence rates based on a comparison of the Depression, Anxiety and Stress Scale (DASS) and the hospital, Anxiety and Depression Scale (HADS).. BMC Psychiatry.

[16] Bjelland I., Dahl A.A., Haug T.T., Neckelmann D. (2002). The validity of the Hospital Anxiety and Depression Scale. An updated literature review.. J. Psychosom. Res..

[17] Fábregas B.C., Vitorino F.D., Rocha D.M., Moura A.S., Carmo R.A., Teixeira A.L. (2012). Screening inventories to detect depression in chronic hepatitis C patients.. Gen. Hosp. Psychiatry.

[18] Crawford J.R., Henry J.D., Crombie C., Taylor E.P. (2001). Normative data for the HADS from a large non-clinical sample.. Br. J. Clin. Psychol..

[19] Henry J.D., Crawford J.R. (2005). The short-form version of the Depression Anxiety Stress Scales (DASS-21): construct validity and normative data in a large non-clinical sample.. Br. J. Clin. Psychol..

[20] Australian Bureau of Statistics (2008).

[21] Loftus E.V., Guérin A., Yu A.P., Wu E.Q., Yang M., Chao J., Mulani P.M. (2011). Increased risks of developing anxiety and depression in young patients with Crohn’s disease.. Am. J. Gastroenterol..

[22] Lane D., Carroll D., Ring C., Beevers D.G., Lip G.Y. (2002). The prevalence and persistence of depression and anxiety following myocardial infarction.. Br. J. Health Psychol..

[23] Kraus M.R., Schäfer A., Faller H., Csef H., Scheurlen M. (2003). Psychiatric symptoms in patients with chronic hepatitis C receiving interferon alfa-2b therapy.. J. Clin. Psychiatry.

[24] Mikocka-Walus A.A., Turnbull D.A., Moulding N.T., Wilson I.G., Holtmann G.J., Andrews J.M. (2008). Does psychological status influence clinical outcomes in patients with inflammatory bowel disease (IBD) and other chronic gastroenterological diseases: an observational cohort prospective study.. Biopsychosoc. Med..

[25] Bjerkeset O., Nordahl H.M., Larsson S., Dahl A.A., Linaker O. (2008). A 4-year follow-up study of syndromal and sub-syndromal anxiety and depression symptoms in the general population: the HUNT study.. Soc. Psychiatry Psychiatr. Epidemiol..

[26] Ickovics J.R., Hamburger M.E., Vlahov D., Schoenbaum E.E., Schuman P., Boland R.J., Moore J., HIV Epidemiology Research Study Group (2001). Mortality, CD4 cell count decline, and depressive symptoms among HIV-seropositive women: longitudinal analysis from the HIV Epidemiology Research Study.. JAMA.

[27] Lyketsos C.G., Hoover D.R., Guccione M., Dew M.A., Wesch J., Bing E.G., Treisman G.J. (1996). Depressive symptoms over the course of HIV infection before AIDS.. Soc. Psychiatry Psychiatr. Epidemiol..

[28] Banovic I., Gilibert D., Cosnes J. (2010). Crohn’s disease and fatigue: constancy and co-variations of activity of the disease, depression, anxiety and subjective quality of life.. Psychol. Health Med..

[29] Kaptein K.I., de Jonge P., van den Brink R.H., Korf J. (2006). Course of depressive symptoms after myocardial infarction and cardiac prognosis: a latent class analysis.. Psychosom. Med..

[30] Smolderen K.G., Aquarius A.E., de Vries J., Smith O.R., Hamming J.F., Denollet J. (2008). Depressive symptoms in peripheral arterial disease: a follow-up study on prevalence, stability, and risk factors.. J. Affect. Disord..

[31] Smedstad L.M., Vaglum P., Moum T., Kvien T.K. (1997). The relationship between psychological distress and traditional clinical variables: a 2 year prospective study of 216 patients with early rheumatoid arthritis.. Br. J. Rheumatol..

[32] Norton S., Sacker A., Young A., Done J. (2011). Distinct psychological distress trajectories in rheumatoid arthritis: findings from an inception cohort.. J. Psychosom. Res..

[33] Fichter M.M., Quadflieg N., Fischer U.C., Kohlboeck G. (2010). Twenty-five-year course and outcome in anxiety and depression in the Upper Bavarian Longitudinal Community Study.. Acta Psychiatr. Scand..

[34] Richards D. (2011). Prevalence and clinical course of depression: a review.. Clin. Psychol. Rev..

[35] Wetherell J.L., Gatz M., Pedersen N.L. (2001). A longitudinal analysis of anxiety and depressive symptoms.. Psychol. Aging.

[36] Grace S.L., Abbey S.E., Pinto R., Shnek Z.M., Irvine J., Stewart D.E. (2005). Longitudinal course of depressive symptomatology after a cardiac event: effects of gender and cardiac rehabilitation.. Psychosom. Med..

[37] Ghany M.G., Nelson D.R., Strader D.B., Thomas D.L., Seeff L.B., American Association for Study of Liver Diseases (2011). An update on treatment of genotype 1 chronic hepatitis C virus infection: 2011 practice guideline by the American Association for the Study of Liver Diseases.. Hepatology.

